# Single- and Two-Phase Flow Characterization Using Optical Fiber Bragg Gratings

**DOI:** 10.3390/s150306549

**Published:** 2015-03-17

**Authors:** Virgínia H.V. Baroncini, Cicero Martelli, Marco José da Silva, Rigoberto E.M. Morales

**Affiliations:** 1 Graduate School of Electrical Engineering and Computer Science, Federal University of Technology-Paraná (UTFPR), Curitiba 80230-901, Brazil; E-Mails: virginia@utfpr.edu.br (V.H.V.B.); mdasilva@utfpr.edu.br (M.J.S.); 2 Graduate School of Mechanical and Materials Engineering, Federal University of Technology-Paraná (UTFPR), Curitiba 80230-901, Brazil; E-Mail: rmorales@utfpr.edu.br

**Keywords:** two-phase flow, single-phase flow, strain sensor, optical fiber Bragg grating

## Abstract

Single- and two-phase flow characterization using optical fiber Bragg gratings (FBGs) is presented. The sensor unit consists of the optical fiber Bragg grating positioned transversely to the flow and fixed in the pipe walls. The hydrodynamic pressure applied by the liquid or air/liquid flow to the optical fiber induces deformation that can be detected by the FBG. Given that the applied pressure is directly related to the mass flow, it is possible to establish a relationship using the grating resonance wavelength shift to determine the mass flow when the flow velocity is well known. For two phase flows of air and liquid, there is a significant change in the force applied to the fiber that accounts for the very distinct densities of these substances. As a consequence, the optical fiber deformation and the correspondent grating wavelength shift as a function of the flow will be very different for an air bubble or a liquid slug, allowing their detection as they flow through the pipe. A quasi-distributed sensing tool with 18 sensors evenly spread along the pipe is developed and characterized, making possible the characterization of the flow, as well as the tracking of the bubbles over a large section of the test bed. Results show good agreement with standard measurement methods and open up plenty of opportunities to both laboratory measurement tools and field applications.

## 1. Introduction

The simultaneous stream of liquid and gas in a pipe, known as gas-liquid two-phase flows, occurs in many industrial applications, such as chemical, oil and nuclear industries. These flows have been an important field of study for physics and mechanical engineering. Therefore, there has been much effort given by the scientific community and industry for the development of monitoring solutions for such conditions, since they are very common and have a large economic impact regarding equipment usage, as well as production efficiency [1]. Two main issues limit the understanding of real-world large-scale problems. First is the difficulty of scaling down flow problems into small laboratory models. For instance, if a several kilometers-long pipeline that is 17” in diameter is transformed into a laboratory-scale pipeline in terms of length, the diameter of the tubes would be on the order of microns or less, unless the density of the fluid is changed, which is also extremely complicated when one is considering the scale factor of kilometers down to meters. Secondly, the computational demand for solving fundamental equations of real-world-sized systems is extremely large, and most of the time, it just cannot be done. Although some researchers have produced successful solutions to engineering two-phase flow problems, the accuracy limits and the general applicability of simulations depend intrinsically on empirical relationships obtained in experiments. Several solutions based on capacitive/resistive sensors [2], gamma-ray densitometry [3], particle imaging velocimetry [4] and wire-mesh sensor technology [5] have been presented, and some are commercially available.

Optical fiber sensors make use of many of the advantages that come from silica optical fibers, which have made this technology suitable for a wide range of environment applications. They are typically small in size, electrically and chemically passive, immune to electromagnetic interference, robust to harsh environments and have a capability to perform high resolution distributed sensing. They have the down side, however, of the need to be in direct contact with the physical quantity been measured, which sometimes turns them into an intrusive sensor. For this specific application, the sensor is intrusive, but it impacts the measurement or the monitored phenomena is negligible, provided its quite small diameter. Given that optical fibers were first developed for communication systems, optical fiber sensors benefit from the important possibility of having links in communications, such as high, dense multiplexing, and so on. Optical fiber Bragg grating sensors are increasingly being used in specific applications in aeronautics, the automotive industry, structure monitoring in civil engineering and undersea oil exploration [6,7,8]. A range of parameters are being measured, such as strain [9], force [10], pressure [11], displacement [12], temperature [13], humidity [14] and radiation dose [15]. For flow measurement, some researchers have applied optical fiber sensors for flow rate measurement in the past [16,17,18], which were basically limited to single-phase flows. More recently, researchers have presented studies employing FBGs for two-phase flow detection in microchannels [19], as an anemometer with a silver-coated FBG [20] and for the measurement of flow the rate and direction using a cantilever [21].

In this paper, an alternative technique is proposed using optical fiber Bragg grating strain sensors to detect single- and two-phase flow problems. The optical fiber gratings are placed transversally to the flow and are subjected to the forces originated by the flowing fluid. As the liquid flows through the optical fiber, it induces a mechanical force along the fiber, which is proportional to the hydrodynamic pressure and, consequently, to liquid flowing mass. As the fiber is subjected to the flow-induced force, the optical fiber Bragg grating wavelength shifts proportionally. For the single-phase flows, the flow velocity is determined basically by the strain measured by the gratings, since the fluid density is constant and well known. In the case of a two phase flow, the speed of the flow is determined by using two gratings positioned at fixed distances between each other, as demonstrated below in the paper. As this technique is based on the property of the flowing fluid, it is possible that the two-phase flow might be characterized by a smaller number of sensors than is necessary with conventional techniques. The capacity of multiplexing tens to hundreds of optical fiber sensors along the pipe is also another advantage of the proposed technique. This allows the characterization of the flow properties, including air bubble and liquid slug velocity, size and shape and their changes as they flow over the pipe length.

## 2. Sensor Unit and Experimental Setup

**Figure 1:**
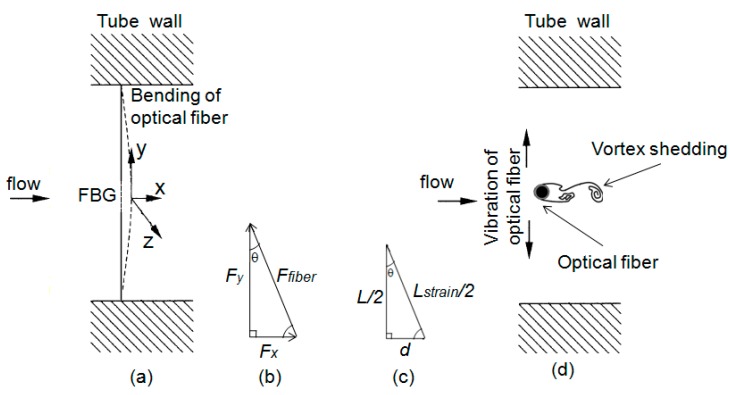
Schematic drawing of the forces that appear in the optical fiber inserted across a flow: (**a**) side view; (**b**) force diagram; (**c**) diagram showing the changes in dimension of the strained optical fiber; and (**d**) top view of the acting forces. FBG, fiber Bragg grating.

The concept of the sensing element consists of an optical fiber Bragg grating positioned transversely to the flow and fixed in the pipe walls. After several experimental tests, it was concluded that the best assembly condition, as well as the best sensor response were achieved when the grating was glued to the pipe walls without any tension. As the fluid flows around the grating, it shifts its wavelength, and the dynamic pressure of the flow is detected. The strain induced in the optical fiber is also a resultant effect of bending displacement and vibration, as shown in Figure 1a,d.

The frequency of such vibration depends on the force exerted by the flow onto the fiber optics and also the density of the fluid, *i.e.*, it depends on the local Reynold’s number. The force field acting on the optical fiber is transverse at the fiber, resulting in changes in the Bragg wavelength. These changes will allow the determination of the force of the flow acting on the fiber. The force field in the horizontal direction (x-axis) is obtained by the shift of fiber Bragg gratings in the optical fiber. As a matter of simplification, one can consider that the entire force field generated by the flow over the optical fiber transverse to the flow can be represented by a single force component represented by $F_{x}$.

When an optical fiber is stretched, it suffers a strain, which is proportional to the amplitude of the longitudinal force *Fy =* *εEA* along the fiber length, where *E* is the Young’s modulus of silica fiber and A is the cross-sectional area of the fiber. For an optical fiber subjected to a force $F_{x}$ (see Figure 1b) in the x-direction, a force component on the y-direction also appears, which leads to strain along the fiber. This strain can be measured by the wavelength shift of an FBG recorded in the optical fiber. Therefore, one can use such a wavelength shift to determine the force $F_{x}$ acting on the fiber. Decomposing the force diagram (Figure 1b):

(1) $$F_{y}=F_{fiber}cos\theta \text{ and }F_{x}=F_{y}tg\theta$$

where $F_{Fiber}$ is the force on the fiber. Considering that θ is very small, it is possible to conclude that $F_{y}=F_{fiber}$. In Figure 1c, an approximation of the geometric changes in the fiber as the flow pushes the fiber is presented. *L/2* and *L_strain_/2* correspond to half of the strained fiber and half of the strained fiber, respectively, and *d* is the displacement of the fiber from its unstrained state to the strained position. Considering that:

(2) $$tg\theta =\frac{d}{L/2}$$

(3) $$L_{strain}=L\left(\varepsilon +1\right)$$

(4) ε=ΔL/L

It is possible to determine that:

(5) $$tg\theta =\sqrt{{\left(\varepsilon +1\right)}^{2}-1}$$

Additionally, the force acting on the fiber is:

(6) $$F_{x}=\varepsilon .E.A\sqrt{{\left(\varepsilon +1\right)}^{2}-1}$$

Given that the Bragg wavelength shift as a result of the applied strain, this can be expressed by [20]:

(7) $$\Delta \lambda_{B}=\lambda_{B}\left(1-\rho_{eff}\right)\varepsilon$$

where $\lambda_{B}$ is the FBG Bragg wavelength, $\Delta \lambda_{B}$ is the wavelength shift and $\rho_{eff}$ is an effective strain-optic coefficient [22]. The amplitude of the force field in the horizontal direction (x-axis) obtained by the shift of fiber Bragg gratings in the optical fiber is determined by:

(8) $$F_{x}=\left(\frac{\Delta \lambda_{B}}{\lambda_{B}\left(1-\rho_{eff}\right)}\sqrt{{\left(\frac{\Delta \lambda_{B}}{\lambda_{B}\left(1-\rho_{eff}\right)}+1\right)}^{2}-1}\right).E.A$$

The mass flow (m) can also be derived from the equations above, since it is proportional to the force ($F_{x}$), as shown below:

(9) $$m\approx \frac{F_{x}}{K.v^{2}}$$

where v is the flow velocity and K  is the constant of proportionality, which depends on the optical fiber sensor assembly properties.

**Figure 2:**
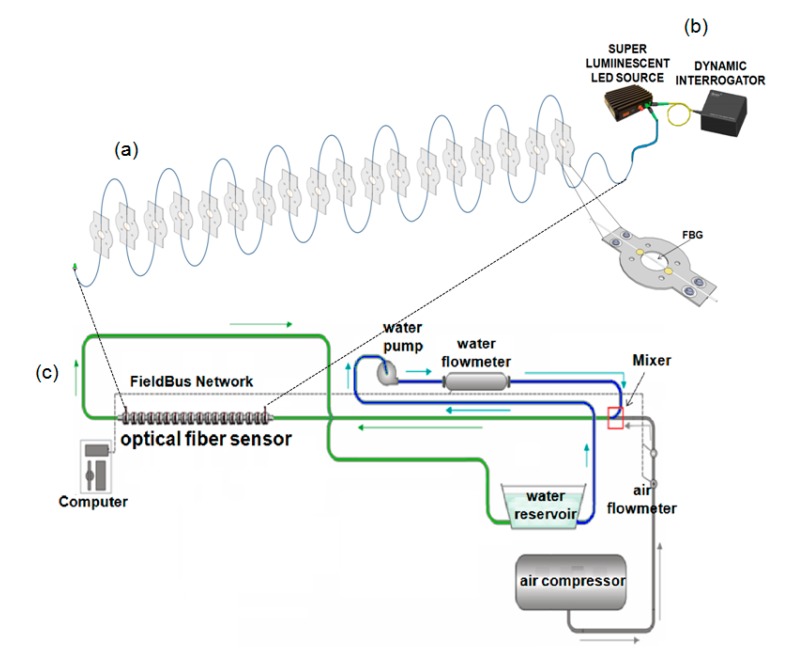
(**a**) Schematic drawing of the optical fiber strain sensors installed in a pipe; (**b**) Measuring setup; (**c**) Experimental rig for simulating different flow regimes.

The sensor setup using optical fiber Bragg gratings is shown in Figure 2b. The fiber Bragg gratings were fabricated using standard single-mode optical fiber (SMF), which was subjected to the 193-nm radiation of an Excimer laser through a phase mask. The spectral response of the FBGs was monitored during the fabrication in order to assure that similar reflectivities for all gratings were achieved. In order to position the FBGs transversally to the flow, metallic plates with V-groove-like channels were fabricated and used to support the gratings, as is shown in Figure 2a. Eighteen gratings multiplexed in wavelength are used in order to allow the monitoring of the flow properties along the length of the test bed.

The sensor unit is connected to an 8 m-long arm of a fluid flow simulation setup (see Figure 2c). A rotating pump is used to generate water flow with rates ranging from 0 to 2.5 m/s, while air from a compressor is mixed with the flowing water, generating the two-phase flow. Water and air flows are both independently computer controlled in order to obtain different flow regimes. The FBG wavelengths were monitored using a commercially-available dynamic interrogator (I-MON 512 USB) and the broadband emission of a super luminescent LED source (DL-BP1-1501A SLED) from Ibsen Photonics Inc., as is schematically represented in Figure 2b. The interrogator was operated with a repetition rate of 800 Hz.

## 3. Results and Discussion

As introduced above, the monitoring of single- and two-phase flows is of great importance to both academic and industrial applications. The latter is because of the ever growing need for controlling and supervising production plants. The first is related to the lack of understanding of many distinct flow conditions, as well as the limitations of the mathematical simulation tools. Experimental measurements can be used to produce design relations, through simple mathematical correlations and establish relationships between the measured variables. In the following, the sessions results of the proposed sensing technique will be presented for single-phase flow of water and a two-phase flow of water and air.

### 3.1. Single-Phase Flow Measurements

**Figure 3:**
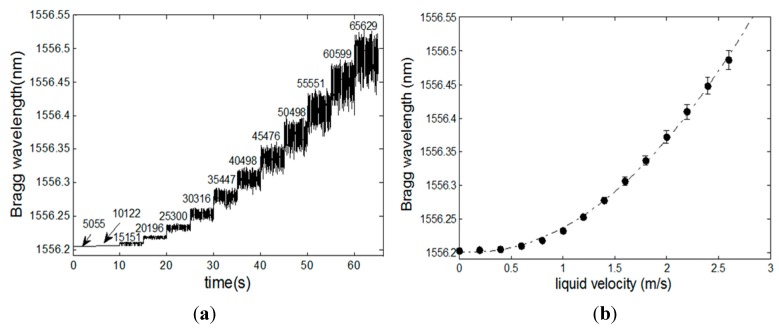
(**a**) Behavior of an FBG strain for single-flow and increasing velocities; the Reynold’s number increases, leading to increasing high frequency oscillations induced on the optical fiber sensor; (**b**)Typical optical fiber Bragg grating wavelength shift and standard deviation against single-phase flow liquid velocity.

In Figure 3, the measurement of single-phase flows for different velocities is presented. Figure 3a shows the effect of the flow velocity on the optical fiber sensor. The time series corresponds to a measurement where the velocity for every segment of the plot is kept constant for approximately 5 s, and it is increased in steps of 0.2 m/s, in order to observe the increase in the signal oscillation as a function of the local Reynold’s number. Two phenomena are observed: (1) the quadratic dependence of the strain against the velocity; (2) an increase of the signal noise, which results from the rising of the local Reynold’s number. Figure 3b shows the effect of the force created by the fluid at distinct speeds resulting in the Bragg wavelength shift on the optical fiber.

This means that it is possible to measure both the speed of the flow, as well as the mass that is flowing through the pipe once the density of the fluid is known. To find the velocity of the liquid measured by the FBG, one can determine the inverse function of the quadratic relationship between the Bragg wavelength and the fluid velocity by the flowmeter.

**Figure 4:**
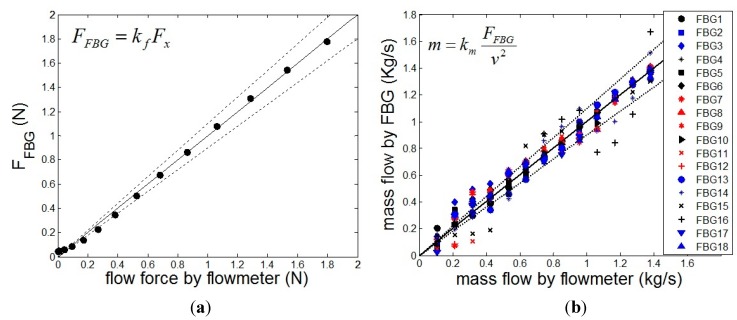
(**a**) Measurement of the flow-induced force to the optical fiber Bragg grating against the flow force determined by the flow properties; (**b**) Measurement of the mass flow for all 18 sensors, indicating the good agreement between the FBG and standard mass flow meter. In both plots, the solid line is at 45°, indicating the position where all values should converge, and the dashed lines indicate a ±10% error.

The comparison between the FBG measured mass flow against the value determined by the standard sensor presented in the test bed is shown in Figure 4. The reference sensor consists of a commercially-available Coriolis sensor, model TCM-230K, manufactured by Tricor, which determines the water flow that can further be transformed into mass flow by using the water density.

The performance of the 18 sensors distributed along the pipe against a reference sensor is shown in Figure 4, and overall, all of the sensors operate with the minimum deviation. Small differences are noted in Sensors 15 and 16, for instance, which are due to problems in the manufacturing, including gluing and alignment.

### 3.2. Two-Phase Flow Measurements

**Figure 5:**
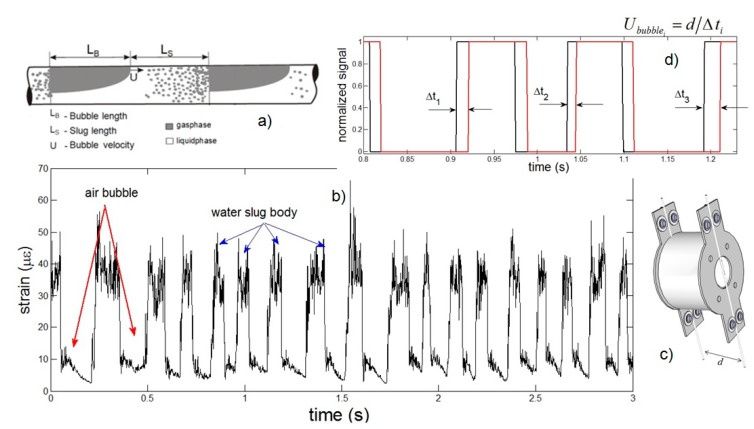
(**a**) Schematic representation of the slug flow with detailed information about the air bubble and liquid slug length and shape; (**b**) Example of a time series detected by the FBG for a typical slug flow for water and air velocities of 2 m/s; (**c**) Detail of the sensor assembly showing a pair of sensors and the fixed distance *d* = 5 cm between them; (**d**) Treated signal for two FBGs as in (c), indicating the time lag Δt between the measured signals that is used to measure the bubble velocity.

The most common flow patterns in horizontal flows are known as dispersed bubble, slug flow, stratified flow and annular flow [1]. Among these, the slug flow occurs in a wide range of flow rates of liquid and gas. The slug flow is characterized by the succession of two distinct regions: the slug, which consists of a liquid column, and an elongated bubble, as shown in Figure 5a.

**Figure 6:**
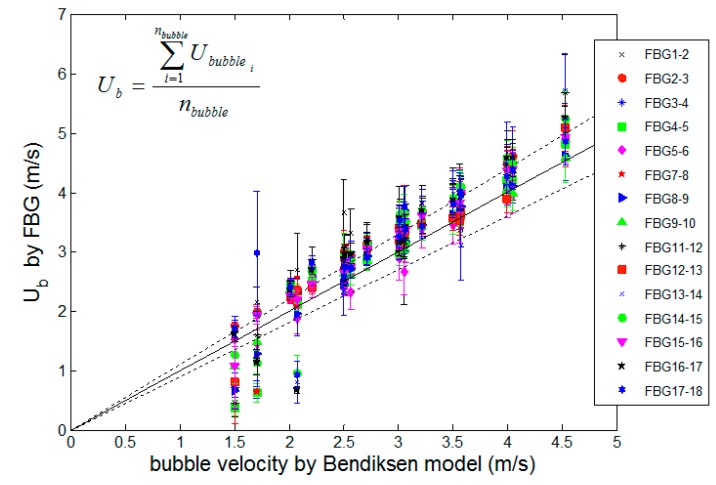
Bubble velocity measurement, using 18 fiber Bragg gratings sensors.

The raw data measured by the optical fiber Bragg grating sensors is shown in Figure 5b. When there is only water passing through the optical fiber sensor, the signal amplitude indicates a maximum load being applied to the fiber. The presence of a bubble decreases the volume of water in the tube cross-section, subsequently decreasing the amplitude of the forces on the fiber sensor, which results in the relaxation of the strain in the fiber, reaching a minimum value. The noisy-like oscillation observed at the higher strain values is possibly originated from the turbulence in the flow. This oscillation is the same as observed in Figure 3a, where the flow velocity increases, as well as the Reynold’s number and, thus, the oscillation induced on the optical fiber sensor. The comparison for all distinct liquid and gas speeds is presented in Figure 6. The x-axis indicates the theoretical expected values for the bubble speed considering Bendiksen’s model, which takes into account the liquid and the gas flow injected into the main pipe of the test bed [23].

## 4. Conclusions

This paper has covered a novel sensing technique to characterize single and two-phase flows. The use of FBG strain sensors as an alternative solution to directly measure the mass flow of the single-phase flow of liquids in pipes is demonstrated. The technique proposed here can be used with virtually any liquid. The operating mechanism of the sensors is based on the measurement of the strain induced by the flowing fluid over the optical fiber. It was also shown that optical fiber sensors based on optical fiber Bragg gratings are particularly suitable for applications where the characterization of the bubble or slug profiles over the pipe length is needed. The sensing technique demonstrated here brings new opportunities for the detection and characterization of single- and dual-phase flows, and it is envisaged that with the correct packaging, this sensor can readily be implemented in real-world problems, where small, intrusive sensors can be used. This includes all sorts of small diameter pipelines present in the chemical sector, for instance. The optical fiber Bragg grating sensors consist, therefore, of an interesting technology, which is minimally intrusive and can be used to generate various information about the sensor with a minimum number of sensors.
